# Estimation of genomic and mitochondrial DNA integrity in the renal tissue of mice administered with acrylamide and titanium dioxide nanoparticles

**DOI:** 10.1038/s41598-023-40676-7

**Published:** 2023-08-19

**Authors:** Hanan R. H. Mohamed, Loren S. T. Behira, Ayman Diab

**Affiliations:** 1 Zoology Department Faculty of Science, Cairo University, Giza, Egypt; 2 Faculty of Biotechnology, October University for Modern Sciences and Arts, 6th of October City, Egypt

**Keywords:** Genetics, Molecular biology, Natural hazards, Health care

## Abstract

The Kidneys remove toxins from the blood and move waste products into the urine. However, the accumulation of toxins and fluids in the body leads to kidney failure. For example, the overuse of acrylamide and titanium dioxide nanoparticles (TiO_2_NPs) in many food and consumer products increases human exposure and risks; however, there are almost no studies available on the effect of TiO_2_NPs coadministration with acrylamide on the integrity of genomic and mitochondrial DNA. Accordingly, this study was conducted to estimate the integrity of genomic and mitochondrial DNA in the renal tissue of mice given acrylamide and TiO_2_NPs. To achieve this goal, mice were administrated orally TiO_2_NPs or/and acrylamide at the exposure dose levels (5 mg/kg b.w) and (3 mg/kg b.w), respectively, five times per week for two consecutive weeks. Concurrent oral administration of TiO_2_NPs with acrylamide caused remarkable elevations in the tail length, %DNA in tail and tail moment with higher fragmentation incidence of genomic DNA compared to those detected in the renal tissue of mice given TiO_2_NPs alone. Simultaneous coadministration of TiO_2_NPs with acrylamide also caused markedly high elevations in the reactive oxygen species (ROS) production and p53 expression level along with a loss of mitochondrial membrane potential and high decreases in the number of mitochondrial DNA copies and expression level of β catenin gene. Therefore, from these findings, we concluded that concurrent coadministration of acrylamide with TiO_2_NPs augmented TiO_2_NPs induced genomic DNA damage and mitochondrial dysfunction through increasing intracellular ROS generation, decreasing mitochondrial DNA Copy, loss of mitochondrial membrane potential and altered p53 and β catenin genes expression. Therefore, further studies are recommended to understand the biological and toxic effects resulting from TiO_2_NPs with acrylamide coadministration.

## Introduction

The incredible rapid increase in industrial progress and various human activities have led to heavily discharge of many chemicals into the environment and increasing their concentration above the natural background levels along with frequent human exposure to chemical pollutants such as heavy metals, acrylamide, and various nanoparticles through various ways such as food and water, threatening human life^1,2^.

Acrylamide is a thermal pollutant formed during heating processing of proteins and carbohydrates rich food at high temperatures, for example during cooking of potatoes and other food products rich in carbohydrates and poor in proteins (cakes, breads and chips) at a temperature higher than 120 °C. Heated carbohydrates and proteins rich foods found to contain about 5–50 mg/kg of acrylamide as well as drinking water contains about 0.5 mg/L^3^. Accordingly, human heavily exposed to acrylamide from eating fried potato products, bread and biscuits, as well as, excessive drinking of coffee increase human intake of acrylamide^4,5^.

The toxicity of acrylamide has been demonstrated in numerous in vitro and in vivo studies. Acrylamide reacts with biological moieties including nucleic acids, proteins, and other large molecules, causing neurotoxic, carcinogenic and reproductive toxic effects^6–10^. Genotoxicity, clastogenicity and mutagenicity of acrylamide have been proven by high elevations in frequency of chromosomal abnormalities, DNA damage and mutations observed after acrylamide treatment^5,10–13^. The study of Alzahrani^14^, also found that the oral acrylamide single administration (10, 20, or 30 mg/kg or repeated administration (10 mg/kg) for one and two weeks significantly increased the frequency of micronuclei and chromosomal abnormalities induction compared to the negative control values.

Unfortunately, humans are exposed to many other chemicals such as nanoparticles together with acrylamide. Titanium dioxide nanoparticles (TiO_2_NPs) are one of the widest used nanoparticles in many food, medical and consumer products such as pharmaceuticals, toothpastes, sweets, chewing jam and cosmetics^15^, as well as, nanoparticles are extensively used in wastewater treatment to remove harmful and xenobiotic chemicals due to their corrosion resistance and high stability^16,17^. All these applications along with the high uses of TiO_2_NPs in increasing crop yield by accelerating photosynthesis of and increasing agricultural productivity specific crops^15^ increase human exposure to TiO_2_NPs and require studying the effect of exposure of nanoparticles on human health.

Upon ingestion of pharmaceuticals, food, water and other consumer products containing TiO_2_NPs nanoparticles such as chewing gums, candies, sweets and artificial flavors, the TiO_2_NPs can diffuse throughout the circulation and enter cells of various organs by endocytosis or/and free diffusion causing gastric toxicity, hepatotoxicity, neurotoxicity and nephrotoxicity^18–22^. Nano-TiO_2_ particles can also directly or indirectly interact with genomic DNA disrupting the integrity of genomic DNA through induction of chromosomal aberrations, DNA breakages, mutations and alterations of apoptotic genes expressions in various experimental models^19,20,22–25^.

As seen, all of the above studies demonstrated toxicity after exposure to acrylamide or TiO_2_NPs separately and almost no studies have investigated the genotoxicity of both acrylamide and TiO_2_NPs although human exposure to acrylamide and TiO_2_NPs together can be done simultaneously. Therefore, the current focused on studying the effect of concurrent simultaneous administration of TiO_2_NPs and acrylamide on genomic and mitochondrial DNA integrity in the kidney tissues of mice. Genomic DNA integrity was assessed using alkaline comet and laddered DNA fragmentation assays, while, mitochondrial membrane potential and level of intracellular reactive oxygen species (ROS) were studied using Rhodamine and 2,7 dichlorofluorescin diacetate dyes, respectively. Moreover, quantitative real time polymerase chain reaction (qRT-PCR) was done to measure the number of mitochondrial DNA copy and expression level of apoptotic genes.

## Materials and methods

### Animals

Swiss Webster male mice weighting 20–25 gm and aging 10–12 weeks were purchased from Animal House of the National Organization for Drug Control and Research (NODCAR). For acclimatization mice were kept in the animal house conditions at the Department of Zoology, Faculty of Science Cairo University under standard dark/light cycle and supplied with standard diet pellets and water that were given ad libitum.

### Chemicals

Acrylamide and TiO_2_NPs were purchased as white powder from Sigma-Aldrich Chemical Company (St. Louis, MO, USA). Powder of TiO_2_NPs < 100 nm was immediately suspended in deionized distilled water prior to use for preparing the given dose of 5 mg/kg b.w equivalent to the human exposure dose^26,27^, while, acrylamide was dissolved in deionized distilled water to prepare the tested dose of 3 mg/kg b.w equivalent to the human exposure dose^3^. Other used chemicals and reagents used in the experiments of this study were of analytical and molecular biology grade.

### Characterization of TiO_2_NPs

Powder of TiO_2_NPs purchased from Sigma-Aldrich Chemical Company (St. Louis, MO, USA) with CAS number 13463-67-7 and a purity 99.5% has been characterized in recent study^13^ using X-ray diffraction (XRD) to ensure the purity of the TiO_2_NPs crystallites and transmission electron microscopy (TEM) to detect the shape and average particle size of suspended TiO_2_NPs in aqueous media.

### Ethical consideration and treatment schedule

The experimentations and study plan were reviewed and approved by the Institutional Animal Care and Use Committee (IACUC) at Cairo University with accreditation number (CU/I/F/15/18). This study was reported according to ARRIVE guidelines and also Animal handling and experimentations were conducted in accordance with the Guidelines of the National Institutes of Health (NIH) regarding the care and use of animals for experimental procedures.

Twenty-four male mice were acclimated for one week and then randomly divided into four groups, six animals per group. The negative control group (group I) was orally given distilled deionized water and the three treated groups (Groups II to IV) were orally administered acrylamide at a dose level of 3 mg/kg body weight^3^ or/and TiO_2_NPs at a dose level of 5 mg/kg body weight^26,27^ separately or simultaneously five times per week for two successive weeks. Mice from all groups were sacrificed 24 h after the last administration and dissected. The obtained kidney tissues were stored at − 80 °C for further molecular studies.

### Estimation of genomic DNA integrity

To assess the effect of acrylamide or/ and TiO_2_NPs administration on the integrity of genomic DNA laddered DNA fragmentation and alkaline comet assays were conducted in the renal tissues of all groups.

### Ladder DNA fragmentation assay

Laddered DNA fragmentation assay was done by gently homogenizing small pieces of renal tissues in cold Tris EDTA (TE) and sodium dodecyl sulfate lysis buffer, RNase A then added and incubating samples for 1 h in lysis buffer at 37 °C. After lysis, proteinase K was added to samples, incubated at 50 °C, and precipitated genomic DNA using cold absolute ethanol. The precipitated genomic DNA was dissolved in deionized dist. water and 15 µl of the dissolved genomic DNA (3 µg) was electrophoresed in 1% agarose gel at 70 V, visualized and photographed using a UV trans-illuminator^28^.

### Alkaline comet assay

According to the protocol described by Tice et al.,^29^ a mixture of renal cell suspension and low melting agarose was spread onto a fully frosted slide pre-coated with normal melting agarose (1%), left to dry and placed in cold lysis buffer (2.5 M NaCl, 100 mM EDTA and 10 mM Tris, pH 10) with freshly added 1% Triton X-100 and 10% DMSO for 24 h in the dark at 4ºC. Slides were incubated in alkaline buffer for 15 min and then denatured single-stranded DNA was electrophoresed at 25 V and 300 mA (0, 90 V/cm) for 30 min. Slides after electrophoresis were neutralized in Trizma base to reanneal denatured single stranded DNA, fixed in cold absolute ethanol, dried and stored at room temperature. Re-annealed double stranded DNA was stained with ethidium bromide, examined and photographed using epi-fluorescent microscope. Fifty nuclei were analyzed for each sample using TriTek Comet ScoreTM Freeware v1.5 scoring software and the three parameters: Tail length, % DNA in tail and tail moment were used as indicators for the DNA breakages in renal cells.

### Estimation of mitochondrial DNA integrity

The impact of acrylamide or/and TiO_2_NPs oral administration on the integrity of mitochondrial DNA was evaluated by studying the mitochondrial membrane potential and measuring the number of mitochondrial DNA copy in renal tissues of all groups.

### Mitochondrial membrane potential

The mitochondrial membrane permeability potential was studied in the renal tissues of control and treated mice with the fluorescent Rhodamine-123 dye^30^. Small pieces of renal tissues were gently homogenized in phosphate buffered saline (PBS), the clear cell suspension was mixed with Rhodamine-123 fluorescent dye and left for 1 h at 37 °C in the dark. Renal cells were then washed twice with PBS and the intensity of fluorescent light emitted from cells stained with Rhodamine-123 imaged and analyzed using an epi-fluorescence microscope at 20 × magnification.

### Mitochondrial DNA copy

To quantify the number of mitochondrial DNA copy in renal tissues of negative control and treated mice, a quantitative real time polymerase chain reaction (RT-PCR) was done: total RNA and DNA was first extracted from renal tissues using GeneJET Purification Kit (Thermo Scientific, USA) and then the RNA was reversely converted into complementary DNA (cDNA) using Oligo(dT)_18_ and Random Hexamer primers according to the manufacturer's instructions for the Revert Aid First Strand cDNA Synthesis Kit (Thermo Scientific, USA). A separate RT-PCR was then conducted in the 7500 Fast system thermal cycler (Applied Biosystem 7500, Clinilab, Egypt) to amplify the mitochondrial 12S ribosomal RNA (12S rRNA) gene using the pre-deigned primers shown in Table 1^31^. The obtained data were standardized against the nuclear 18S rRNA gene as a housekeeping gene and the number of mitochondrial DNA copies was then determined using a comparative Ct (DDCt) method. Results were expressed as mean ± S.D.

**Table 1:** The sequences of used primers.

Gene	Strand	Sequence
12S rRNA	Forward Reverse	5-ACCGCG GTC ATA CGA TTA AC-3 5-CCC AGTTTG GGT CTT AGC TG-3
18S rRNA	Forward Reverse	5-CGC GGTTCT ATT TTG TTG GT-3 5-AGT CGG CATCGT TTA TGG TC-3
*P53*	Forward Reverse	5′-ANCCATCGGAGCAGCCCTCAT-3′ 5′-TACTCTCCTCCCCTCAATAAG-3′
β-Catenin	Forward Reverse	5′GCTGACCTGATGGAGTTGGA-3′ 5′-GCTACTTGCTCTTGCGTGAA-3′
β-actin	Forward Reverse	5′-TCACCCACACTG TGCCCATCT ACG A-3′ 5′-GGATGCCACAGGATTCCATACCCA-3′

### mRNA expression of p53 and β-catenin genes

To measure the mRNA expression level of p53 and β-Catenin genes in the renal tissues of negative control and treated mice; whole RNA was extracted and reversely transcribed into complementary DNA (cDNA) using the GeneJET RNA Purification and Revert Aid First Strand cDNA Synthesis Kits (Thermo Scientific, USA), respectively. For cDNA synthesis each a µg of template RNA and 1 µl of primer were added to a sterile nuclease free water to complete the final volume to 12 µl in nuclease free tube on ice. Then added reaction buffer, RNase Inhibitor, nucleotides and Revert Aid M-MuLV RT completed the final volume to 20 µl. Samples were mixed gently, incubated 42 °C for 60 min and terminated the reaction by heating at 70 °C for 5 min. Amplification of p53 and β-Catenin genes was then conducted using the primers' sequences listed in Table 1 and previously designed by^32,33^. The results obtained from RT-PCR were then normalized to the housekeeping β-actin gene and the comparative Ct (DDCt) method was used to calculate the fold changes in the expression level of p53 β-Catenin genes.

### Generation of intracellular ROS

The generation level of ROS within renal cells was evaluated by mixing renal cells suspension with 2,7 dichlorofluorescin diacetate dye that enters passively. Mixed cells with dye were left in dark for thirty minutes to allow interaction between dye and generated ROS within cells. After incubation cells were spread on a clean slide, visualized and photographed the forming the highly fluorescent dichlorofluorescein compound under epi-fluorescence microscope at 20 × magnification^34^.

### Statistical analysis

Results obtained from Comet assay and RTPCR were analyzed using the Statistical Package for the Social Sciences (SPSS) (version 20) at the significance level p < 0.05. One way analysis of variance (ANOVA) and Duncan's test were done to compare between the negative control and three treated groups. All results this study are expressed as mean ± Standard Deviation (S.D).

## Results

### Characterization of TiO_2_NPs

Characterization of TiO_2_NPs in our recent study^13^ confirmed the purity of purchased TiO_2_NPs powders through the appearance of unique bands for TiO_2_NPs crystals and also demonstrated the polyhedral shape the of well dispersed TiO_2_NPs with about 60 nm median particles size shown in TEM image.

### Integrity of genomic DNA

#### Laddered DNA fragmentation

**Figure 1:**
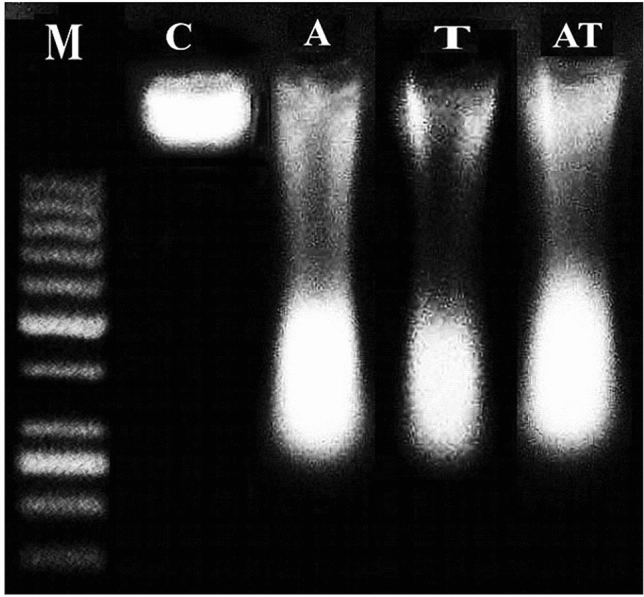
Representative photo for the seen electrophoresed pattern of renal genomic DNA extracted from negative control mice (C) and mice given orally acrylamide (A) and TiO_2_NPs (T) separately or simultaneously together (AT). M: Marker. Three samples were analyzed per each group.

As displayed in Fig. 1 oral administration of TiO_2_NPs (5 mg/kg) and acrylamide (3 mg/kg) separately or simultaneously together caused dramatic damage to renal genomic DNA as displayed by the seen smeared fragmentized pattern of DNA running on agarose gel compared to the intact DNA pattern of the negative control mice. However, the degree of DNA damage observed after administration of TiO_2_NPs alone was lower than that observed after administration of acrylamide alone or in combination with TiO_2_NPs as displayed in Fig. 1).

### Alkaline comet assay

**Figure 2:**
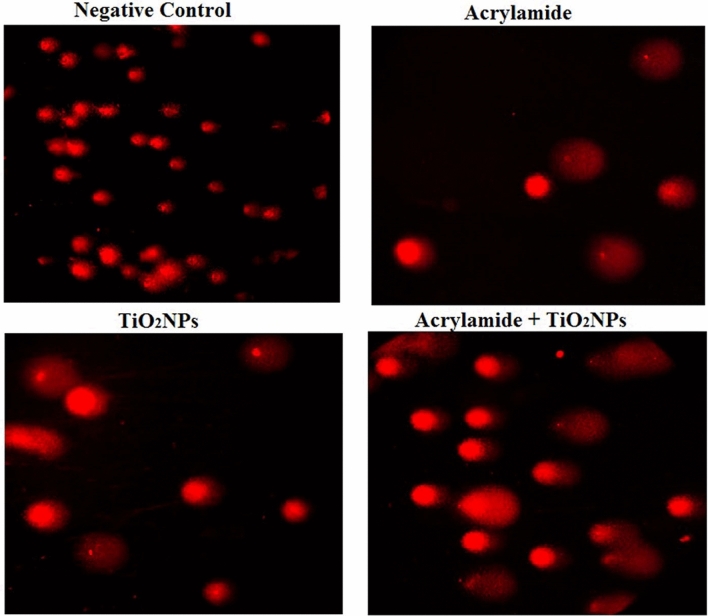
Representative photos for the scored Comet nuclei in the renal cells of the negative control mice and mice given orally acrylamide and TiO_2_NPs separately or simultaneously together.

Results of Comet assay demonstrated the induction of DNA breakages by TiO_2_NPs (5 mg/kg) and acrylamide (3 mg/kg) administered separately or simultaneously together by the statistically significant (p < 0.001) elevations in tail length, %DNA in tail and tail moment noticed in renal tissues of mice given TiO_2_NPs or/and acrylamide compared to the negative control values (Table 2). Otherwise, the DNA damage induced by administration of acrylamide alone or simultaneously with TiO_2_NPs was significantly (p < 0.001) higher than that induced by administration of TiO_2_NPs alone. This was manifested by the statistical significant (p < 0.001) elevations in the three Comet parameters: tail length, %DNA in tail and tail moment observed after administration of acrylamide alone or in combination with TiO_2_NPs compared to their values in renal tissues of mice given TiO_2_NPs alone (Table 2). Examples for the scored Comet nuclei in the renal tissues of the negative control group and mice given acrylamide or/and TiO_2_NPs are displayed in Fig. 2.

**Table 2:** Induction of DNA damage in the renal tissue of negative control mice and mice given orally acrylamide or/and TiO_2_NPs.

Group	Treatment (dose mg/kg)	Tail length (px)	%DNA in tail	Tail moment
I	Negative control (deionized water)	3.22 ± 0.72^a^	14.36 ± 0.73^a^	0.60 ± 0.01^a^
II	Acrylamide (3 mg/kg)	13.51 ± 2.29^b^	30.03 ± 3.00^b^	5.44 ± 0.80^b^
III	TiO_2_-NPs (5mg/kg)	12.26 ± 1.04^b^	23.03 ± 2.01^c^	2.74 ± 0.48^c^
IV	Acrylamide + TiO_2_-NPs	16.51 ± 1.53^c^	32.29 ± 3.62^b^	4.26 ± 0.18^d^
One way analysis of variance		F = 42.66 P < 0.001	F = 29.74 P < 0.001	F = 58.56 P < 0.001

Six mice were used for each group.

Results are expressed as mean ± SD.

Results were analyzed using one-way analysis of variance followed by Duncan’s test to test the similarity between the control and three treated groups.

According to Duncan's test means with different letters indicates statistical significant difference between the compared groups in the same column at a significant level of p < 0.001.

### Integrity of mitochondrial DNA

#### Mitochondrial membrane potential

**Figure 3:**
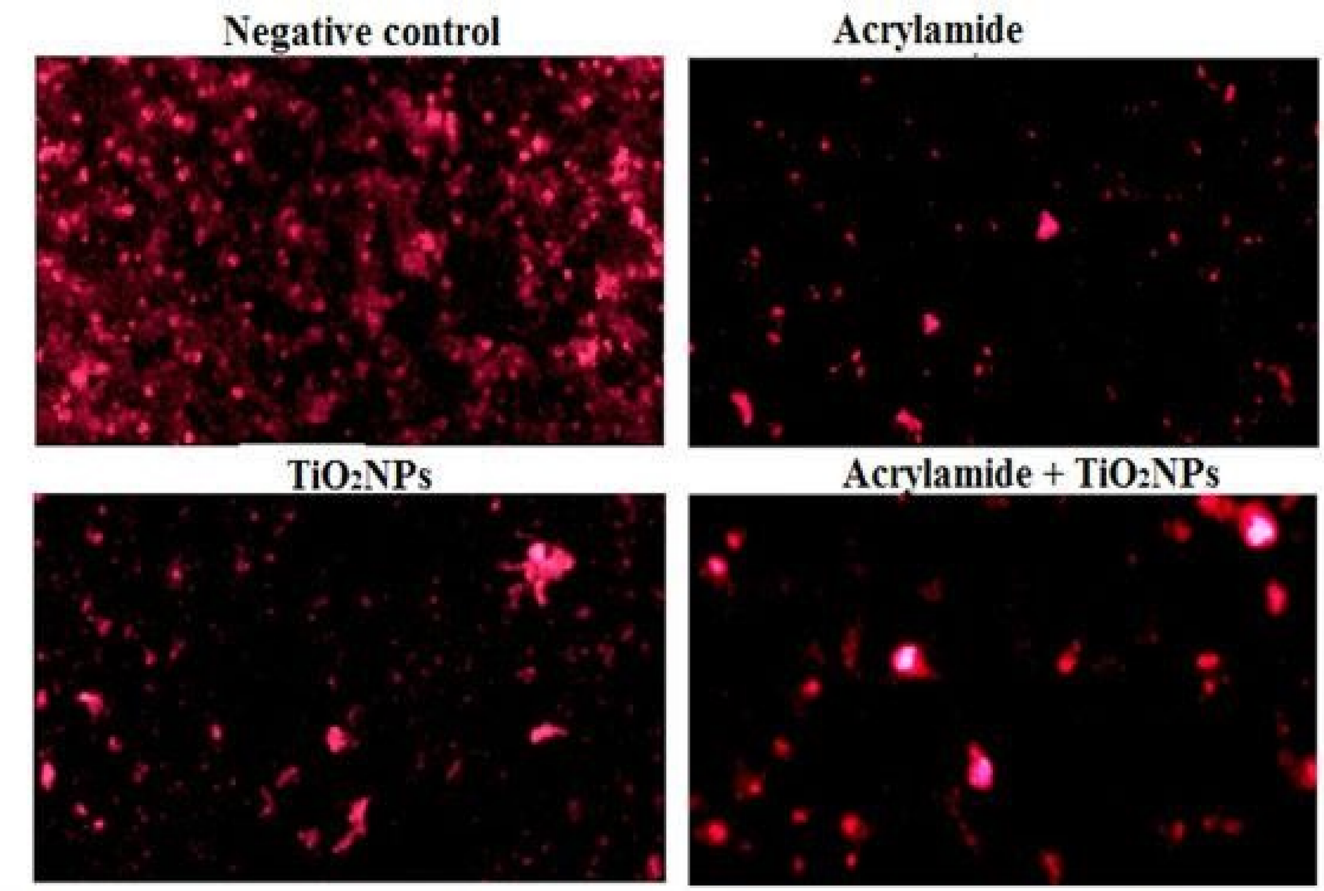
Representative photos for the mitochondrial membrane permeability potential observed in the renal cells of the negative control mice and mice given orally acrylamide and TiO_2_NPs separately or simultaneously together.

Staining the renal cells with Rhodamine-123 dye showed that oral administration of acrylamide and TiO_2_NPs separately or together simultaneously caused a large reduction in the integrity of the mitochondrial membrane permeability potential as manifested by the greater decreases in the intensity of the fluorescence light emitted from the treated renal cells compared with the light emitted from the untreated control renal cells (Fig. 3). Moreover, oral administration of TiO_2_NPs alone caused less reduction in the integrity of mitochondrial membrane potential compared to reductions resulting from administration of acrylamide alone or in combination with TiO_2_NPs (Fig. 3).

### Mitochondrial DNA copy

**Figure 4:**
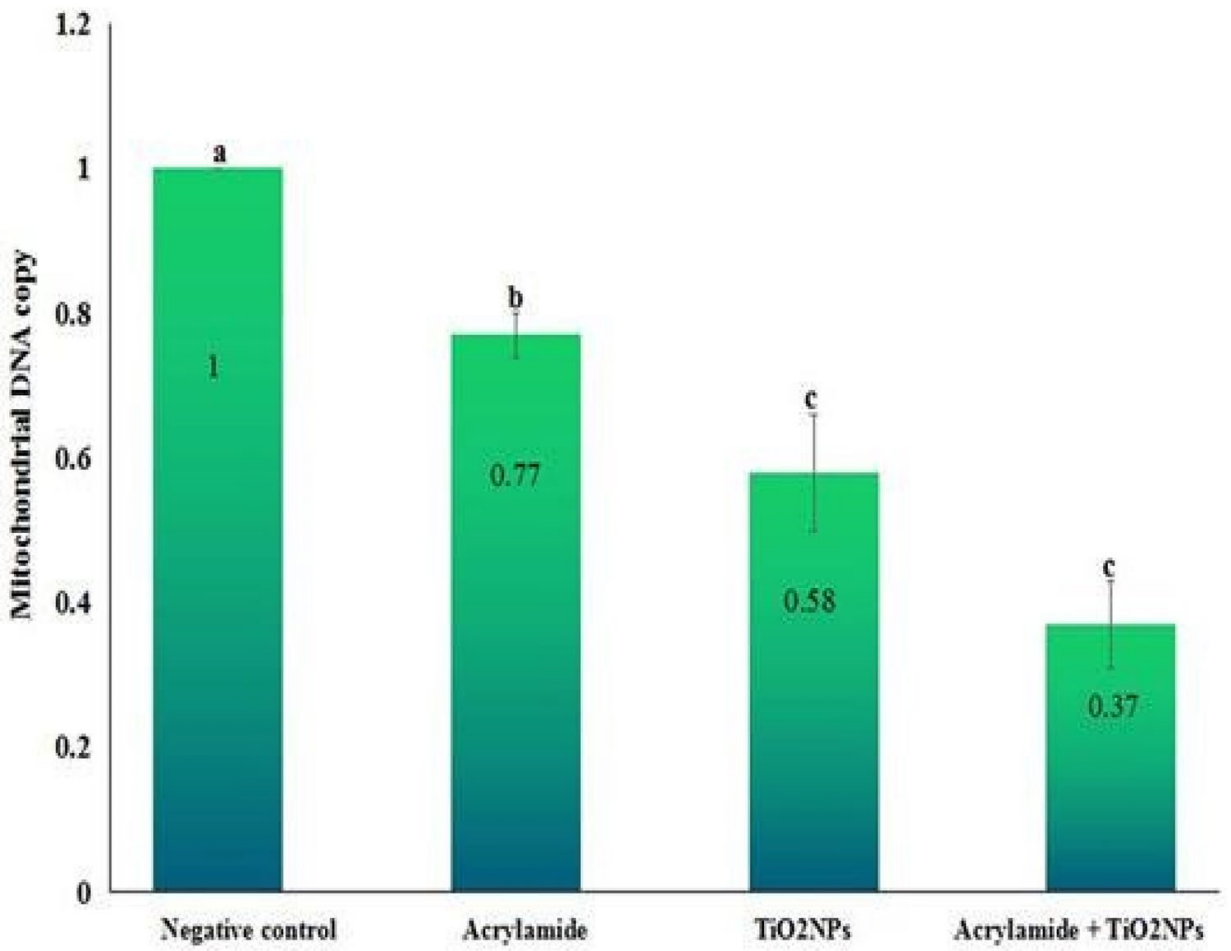
Variation in the copy of mitochondrial DNA in the renal cells of the negative control mice and mice given orally acrylamide and TiO_2_NPs separately or simultaneously together. Six mice were analyzed for each group. Results are expressed as mean ± SD and analyzed using one-way analysis of variance followed by Duncan’s test to test the similarity between the control and three treated groups. Means with different letters indicates statistical significant difference between the compared groups at a significant level p < 0.001.

The copy number of mitochondrial DNA was statistically significantly (p < 0.001) decreased after multiple oral administration of acrylamide or/and TiO_2_NPs compared to the copy of the negative control mitochondrial DNA as seen in Fig. 4. Otherwise, oral administration of acrylamide alone resulted in less reduction in mitochondrial DNA copy than those resulting from administration of TiO_2_NPs alone or in combination with acrylamide (Fig. 4).

### Expression of p53 and β-Catenin genes

As noted in Table 3, multiple oral administration of acrylamide and TiO_2_NPs caused statistical significant (p < 0.001) elevations in the expression level of p53 gene compared with that of the negative control renal tissue. Meanwhile, the expression level of the p53 gene in renal cells of mice orally given TiO_2_NPs alone was significantly (p < 0.001) lower than that expressed in the renal tissues of mice given acrylamide alone or simultaneously with TiO_2_NPs (Table 3).

**Table 3:** Expression level of p53 and β-Catenin genes in the renal tissue of negative control mice and mice given orally acrylamide or/and TiO_2_NPs.

Group	Treatment (dose mg/kg)	β-Catenin	P53
I	Negative control (deionized water)	1.00 ± 0.00^a^	1.00 ± 0.00^a^
II	Acrylamide (3 mg/kg)	0.71 ± 0.06^b^	2.57 ± 0.09^b^
III	TiO_2_-NPs (5mg/kg)	0.81 ± 0.03^c^	1.70 ± 0.04^c^
IV	Acrylamide + TiO_2_-NPs	0.50 ± 0.05^d^	2.48 ± 0.08^d^
One way analysis of variance		F = 73.61 P < 0.001	F = 339.81 P < 0.001

Six mice were used for each group.

Results are expressed as mean ± SD.

Results were analyzed using one-way analysis of variance followed by Duncan’s test to test the similarity between the control and three treated groups.

According to Duncan's test means with different letters indicates statistical significant difference between the compared groups in the same column at a significant level of p < 0.001.

On the contrary, several oral administration of acrylamide and TiO_2_NPs caused statistically significant (p < 0.001) decreases in the expression level of the β-Catenin gene compared to that of the negative control (Table 3). On the other hand, the expression level of β-Catenin gene in renal tissue of mice administered TiO_2_NPs alone was statistically (p < 0.001) higher than that of mice given acrylamide alone or simultaneously with TiO_2_NPs (Table 3).

### Generation of intracellular ROS

**Figure 5:**
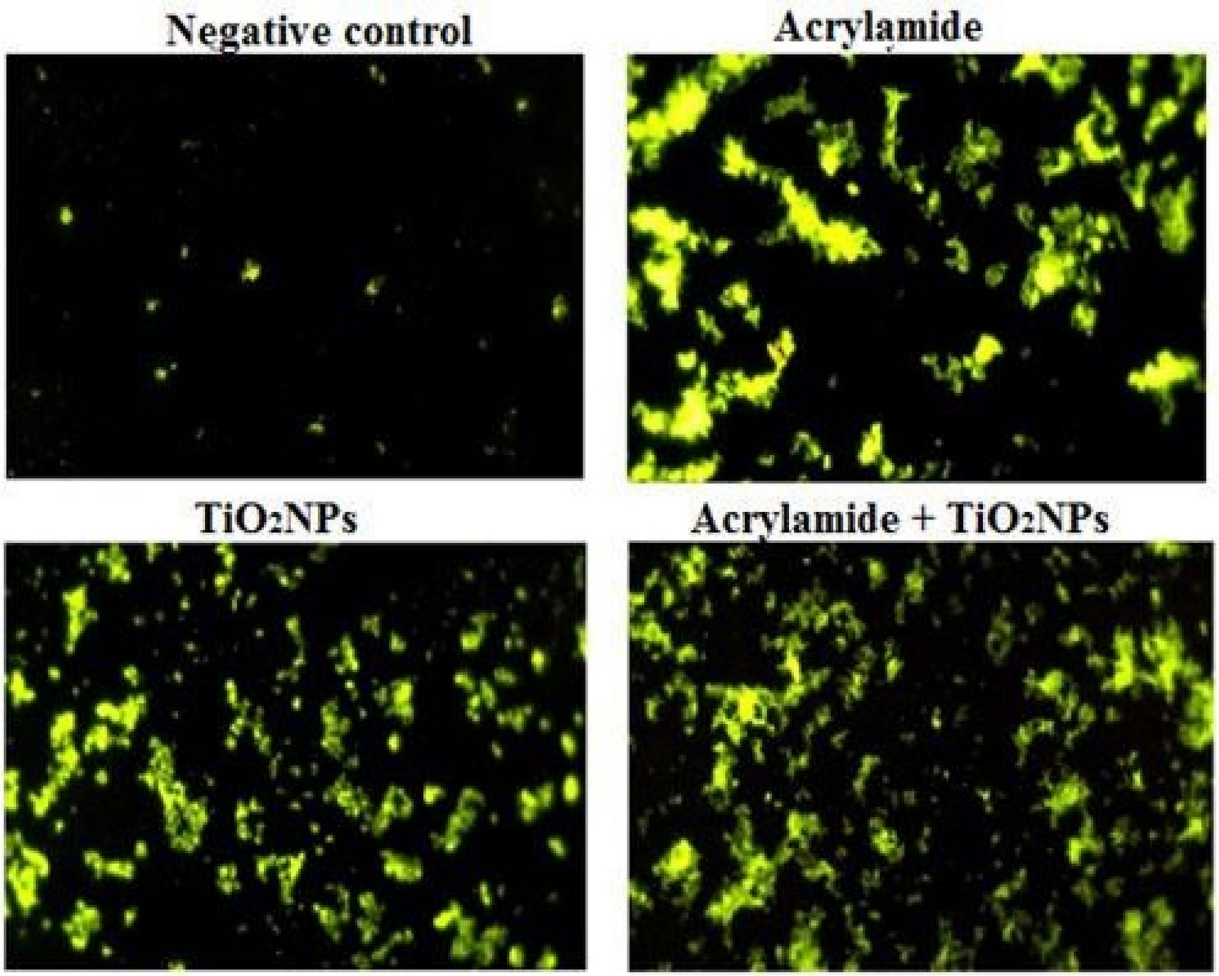
Representative photos for the ROS level generated within the renal cells of the negative control mice and mice given orally acrylamide and TiO_2_NPs separately or simultaneously together.

Examination of renal cells stained with the 2,7 dichlorofluorescin diacetate (DCFH-DA) dye using epi-fluorescent microscope showed that ROS are highly generated after oral administration of TiO_2_NPs or/and acrylamide as the intensity of the fluorescent light emitted from the stained renal cells was remarkably higher compared to those emitted from the negative control renal cells (Fig. 5).

## Discussion

Intensive uses of various consumer and food products containing acrylamide or TiO_2_NPs increase the risk of human exposure to acrylamide TiO_2_NPs together. However, almost no studies have investigated the effect of acrylamide and TiO_2_NPs coadministration on the integrity of genomic and mitochondrial DNA in kidney tissues. Therefore, this study was done to estimate the effect of simultaneous co-administration acrylamide and TiO_2_NPs on the integrity of genomic and mitochondrial DNA in renal tissues of mice.

In this study, acrylamide and TiO_2_NPs in this study were orally administered to mice at the dose level 3 mg/kg and 5 mg/kg, respectively, equivalent to the human exposure dose, because human excessive consumption of high temperature cooked starchy foods and other acrylamide containing foodstuffs reach human daily intake of acrylamide to 3 mg/kg by^3,35,36^. Similarly, the massive use of TiO_2_NPs in plastics, cosmetics toothpaste, Candy, sweets, gums, artificial flavors and other foods due to their high stability and anti-corrosion properties increases human ingestion of a large amount of TiO_2_NPs exceeding 5 mg/kg^26,27,37^.

Our results demonstrated the genotoxicity of acrylamide (3 mg/kg) or/and TiO_2_NPs (5 mg/kg) given orally daily over two weeks through the noticed fragmented smeared pattern of genomic DNA electrophoresed on ethidium bromide stained agarose gel compared to the intact appearance of the negative control genomic DNA. Similarly, the noticed remarkable elevations in measured Comet parameters: tail length, %DNA in tail and tail moment confirmed the induction of DNA breakage after multiple administration of the tested low doses of acrylamide or/and TiO_2_NPs in kidney tissues of mice because alkaline Comet assay sensitively detects single and double stranded DNA breakages^29^. These findings thus supported the demonstrated genotoxicity of acrylamide and TiO_2_NPs administered separately in previous studies^19,20,23,24,38–40^.

The generation of ROS normally occurs within cells during numerous metabolic processes. However, exposure to various toxic agents leads the production of intracellular ROS over the normal level leading to a disruption of the balance between ROS generation and antioxidant defense system inducing oxidative stress^13,41–43^. Excessive generation of ROS by acrylamide or/and TiO_2_NPs was manifested in this study by marked increases in the intensity of fluorescent light emitted from renal cells stained with 2,7 Dichlorofluorescein diacetate dye consistent with the previous suggestion of oxidative stress induction through ROS generation as an accepted mechanism of acrylamide or TiO_2_NPs induced toxicity^13,20,22,44,45^. Therefore, the increased DNA damage induction observed in the renal tissues of mice orally given low doses of acrylamide and TiO_2_NPs together may result from the observed higher ROS generation that attacks cellular proteins, lipids, carbohydrates, and DNA causing cell damage and necrosis making renal cells more susceptible to DNA damage than that induced after administration of acrylamide or TiO2NPs alone. These results are consistent with our recent study showing increased TiO_2_NPs genotoxicity when TiO_2_NPs coadministered with acrylamide through increased ROS generation in mice brain tissue^13^*.*

Oxidizing cellular macromolecules and depleting antioxidants by over ROS generation also promotes mitochondrial DNA damage and triggers apoptosis^46,47^. Our finding of Loss of mitochondrial membrane potential integrity and remarkable reduction in number of mitochondrial DNA copies demonstrated the induction of mitochondrial dysfunction by multiple administrations of the tested low doses of TiO_2_NPs or acrylamide. Moreover, dramatic reduction of mitochondrial DNA copies and impairment of mitochondrial membrane potential noticed in the mice renal cells after simultaneous coadministration of TiO_2_NPs with acrylamide can be attributed to the induced increased extra ROS generations that highly disrupted mitochondrial membrane and mitochondrial DNA integrity hampering mitochondrial DNA biogenesis^43,48,49^.

Apoptosis occurs naturally during aging and development to preserve cell populations in the tissues. However, inappropriate apoptosis can be induced by excessive ROS generation and DNA damage induction^50^. Consequently, our findings on the marked induction of DNA damage, ROS generation and mitochondrial dysfunction after chronic administration of TiO_2_NPs or/and acrylamide may serves as a signal triggering apoptosis of renal cells because even a single double-stranded DNA break is sufficient to induced cellular death^51^. Moreover, the remarkable elevations in the expression level of the apoptotic p53 gene and significant reductions in the expression level of β-Catenin gene noticed after administration of TiO_2_NPs or/and acrylamide confirmed the induction of apoptosis by TiO_2_NPs or/and acrylamide administration because overexpression of the p53 gene mediates and stimulates apoptosis by reducing the expression of β-Catenin as well as accelerated degradation of the β-Catenin protein itself^52,53^.

## Conclusion

Based on the above findings, it was concluded that the coadministration of TiO_2_NPs with acrylamide increased the genomic DNA damage and mitochondrial dysfunction induced by administration of TiO_2_NPs or acrylamide alone by significantly increasing the generation of ROS and the expression of p53 and β-catenin genes. Therefore, further studies are recommended to understand the biological and toxic effects of coadministration of TiO_2_NPs with acrylamide.

## Data Availability

The datasets used and/or analyzed during the current study are available from the corresponding author on reasonable request.
